# Inflammatory Marker sTREM-1 Reflects the Clinical Stage and Respiratory Tract Obstruction in Allergic Asthma Bronchiale Patients and Correlates with Number of Neutrophils

**DOI:** 10.1155/2012/628754

**Published:** 2012-07-05

**Authors:** Maria Bucova, Magda Suchankova, Martin Dzurilla, Mojmir Vrlik, Helena Novosadova, Eva Tedlova, Stefan Urban, Edita Hornakova, Marianna Seligova, Vladimira Durmanova, Peter Penz, Juraj Javor, Ema Paulovicova

**Affiliations:** ^1^Institute of Immunology, Faculty of Medicine, Comenius University, 813 72 Bratislava, Slovakia; ^2^Centre of Immunology, 036 59 Martin, Slovakia; ^3^Institute of Pneumology and Phthisiology, Faculty of Medicine, Comenius University, University Hospital, 813 72 Bratislava, Slovakia; ^4^Institute of Internal Medicine I, Faculty of Medicine, Comenius University, University Hospital, 813 72 Bratislava, Slovakia; ^5^Institute of Chemistry, Slovak Academie of Science, Centre of Excellence Glycomed, 845 41 Bratislava, Slovakia

## Abstract

The knowledge that asthma is an inflammatory disorder has prompted us to investigate the plasma levels of a new inflammatory marker sTREM-1 that is released from the surfaces of activated neutrophils and monocytes. The plasma levels of sTREM-1 were analysed by a sandwich ELISA test in the cohort of 76 patients with allergic asthma bronchiale and 39 healthy controls. Our results revealed more than 3.5 times higher levels of sTREM-1 in AB patients (92.3 pg/mL ± 125.6) compared with healthy subjects (25.7 pg/mL ± 9.2; *P* = 0.0001). Higher levels of sTREM-1 were found also in patients with exacerbated AB (170.5 pg/mL ± 78.2) compared with nonexacerbated AB patients (59.1 ± 78.2; *P* < 0.0001), patients with respiratory tract obstruction (176.4 pg/mL ± 177.8), than those without obstruction (51.99 pg/mL ± 64.0; *P* < 0.0001) and patients with anti-IgE therapy (*P* < 0.0001). Levels of sTREM-1 correlated with number of leucocytes (*P* = 0.002), and absolute number of neutrophils (*P* = 0.001). Elevated plasma levels of sTREM-1 reflect the severity, state of exacerbation, presence of respiratory tract obstruction in AB patients and together with increased number of neutrophils point to the role of neutrophils in inflammation accompanying AB.

## 1. Introduction

TREM-1 (Triggering receptor expressed on myeloid cells), first described in 2000 [1], belongs to a family related to the natural killer cell receptors and is constitutively expressed on the surface of myeloid cells neutrophils, monocytes, and macrophages [2]. Although the biochemical structure of TREM-1 ligands remains to be identified, some studies suggest for inflammatory response products participation. Expression of TREM-1 is upregulated after stimulation with bacterial and fungal products, and also sterile noninfectious stimuli—inflammatory cytokines or other mediators of inflammation, for example, prostaglandin E2 (PGE2) and some endogenous substances released during inflammation—damage-associated molecular patterns [2–6]. HMGB1 (high mobility group box 1 protein) and HSP70 (Heat shock protein) released from necrotic cells function as endogenous danger signals, and TREM-1is involved in mediating proinflammatory responses [7]. Other results [6] provide evidence thatTREM-1may contribute the development of nonmicrobial inflammatory diseases through the enhancement of inflammatory responses [6]. Hypoxia itself modulates the gene expression profile in human mature dendritic cells, and TREM-1 molecule was defined as a novel hypoxic marker *in vitro* and *in vivo* [8]. The involvement of TREM-1 activation in the enhancement of inflammatory response mediated by Toll-like receptor-2 and -4 stimulation was detected [9].

Myeloid cells activation upon a ligand binding to this receptor generally results in inflammatory response amplification, enhanced production of proinflammatory mediators, mainly of tumour necrosis factor-alfa (TNF-*α* and of interleukin-8 (IL-8) [2, 10], increased expression of costimulatory molecules on the surface of macrophages, and enhanced antigen presentation that triggers T-cell proliferation [1, 10]. Neutrophils activated through TREM-1 enhance their respiratory burst activity, degranulation, phagocytosis, release of myeloperoxidase, and IL-8, respectively [11]. TREM-1 by propagating inflammatory responses in inflammatory diseases thus accelerates not only the immune system activation, but also tissue destruction [6]. However, anti-inflammatory cytokines synergistically inhibit its expression [10, 12]. 

The membrane form of TREM-1 can be cleaved from the surface of activated myelocytes by plasma metalloproteinases and its ectodomain is released into microenvironment [13]. This soluble TREM-1 molecule (sTREM-1) could act as adownregulator of inflammation—binding to the natural ligand, sTREM-1 prevents its binding to the membrane TREM-1 and subsequent cell activation [12]. sTREM-1 can be measured in biological fluids and may be useful as a diagnostic tool. The highest levels of sTREM-1 molecules have been found in patients with sepsis [14] and other inflammatory diseases caused mainly by extracellular microorganisms—bacteria and fungi [15–18], as well as inflammatory states of noninfectious origin, for example, rheumatoid arthritis [19, 20].

Knowledge thatasthmais an inflammatory disorder [21] has prompted us to investigate the plasma levels of sTREM-1 in patients suffering from allergic asthma bronchiale.

## 2. Subjects and Methods

76 patients suffering from asthma bronchiale (AB) (32 men, 44 woman), mean age = 37.4 ± 21.4) were enrolled in our study. 28 out of them suffered from a mild intermittent AB, 18 from a mild persistent AB, 14 from a moderate persistent AB, and 16 from a severe persistent AB. Inclusion criteria for asthma stage classification were according the Global Initiative for Asthma (GINA) guidelines [22]. Out of all AB patients, 25 patients were in acute exacerbation (EAB), the rest of them (51) were without acute exacerbation (non-exacerbated AB-NEAB). 30 AB patients suffered from respiratory tract obstruction, 46 were without respiratory tract obstruction. All patients were treated with corticosteroids, 11 patients with severe persistent AB were on anti-IgE monoclonal antibody (Omalizumab) therapy. 

Patients originated from specialized allergo-immunological outpatients' departments. Exacerbated AB patients with moderate persistent and severe persistent AB were in the care of Institute of Pneumology and Phthisiology, Comenius University, University Hospital in Bratislava.

All patients were carefully examined by their physicians, they underwent spirometric examinations, bronchoprovocation test, allergen skin tests, and levels of total IgE were detected too. Height and body mass of patients were measured and the body mass index was calculated for each patients. 

Spirometry was measured by pneumotachograph Masterscope Masterscreen (Jaeger). The examination consisted of minimally three valid forced expiratory maneuvers after amaximum inspiration. Expirium lasted at least 8–10 seconds. The largest forced vital capacity (VC) was taken into account. Reference values were according to publication Kristufek et al. [23]. The presence of airway obstruction was defined according to criteria of the European Respiratory Society [24], when the ratio of forced expiratory volume in one second (FEV_1_) to vital capacity (VC), (FEV_1_/VC), was less than 88% of the reference value in men and less than 89% of the reference value in women. Degrees of obstructive ventilatory disorders were defined according to the decrease of FEV_1_ (forced expiratory volume in one second) as follows: mild degree (mild obstructive ventilatory disorder): 60% of the reference value—lower limit of normal, moderate degree (moderate obstructive ventilatory disorder): 59%–45% of the reference value, and severe degree (severe obstructive ventilatory disorder): less than 45% of the reference value.

We could not always obtain complete results of spirometry from each patient. However, medical reports with diagnostic conclusions of these tests, that is, asthma bronchiale with mild, moderate, or severe degree of obstructive ventilatory disorder, respectively, were available for each patients. These data enabled us to divide patients into two groups. One group represented patients without obstructive ventilatory disorder, the second included patients with mild, moderate, and severe obstructive ventilatory disorder. So, in statistic evaluation, the presence of bronchoconstriction was defined as nominal variable (0-without bronchoconstriction, 1-with bronchoconstriction).

Bronchoprovocation tests were performed with metacholine according to American Thoracic Society [25]. The bronchial hyperreactivity (hyperresponsiveness) was considered when FEV_1_ fell by 20% after metacholine dose of 8 mg/mL (PC_20_ < 8 mg/mL).

The study was approved by the Local Ethical Committee of Faculty of Medicine Comenius University in Bratislava and written informed consent was obtained from all patients.

The total plasma IgE level was determined in all patients by an electrochemiluminescence method (Modular Analytics E170, Roche, USA). Patients were assessed for both total and differential blood cell counts (total numbers of leucocytes, red blood cells, platelets, absolute, and relative number of neutrophils, lymphocytes, monocytes, eosinophils, and basophils by an optic flow cytometry (Sysmex XT 1800i (Sysmex Corporation). Plasma levels of CRP (C-reactive protein) were detected turbidimetrically (Advia 2400, Siemens) directly at the day of blood collection. All aforementioned laboratory examinations were done in central laboratory (Medirex, Ltd., Bratislava).

The control group of healthy subjects represented 39 volunteers (21 men, 18 woman; mean age ± STD = 37.3 ± 13.7). None of these subjects suffered from AB and all of them were without any history of pollen allergy.

Plasma levels of sTREM-1 were investigated by a sandwich Elisa test (Human sTREM-1 ELISA; R&D, Quantikine, Minneapolis, USA), exactly according to instructions of the manufacturer.

## 3. Statistical Analysis

The one-sample Kolmogorov-Smirnov test was used to determine whether the investigated population followed a normal distribution. Either Anova or nonparametric Mann-Whitney *U*-test were used to determine the difference and the statistical significance. The association of sTREM-1 plasma levels with clinical stage of AB was evaluated by Kruskal Wallis test. For correlation analysis of non-parametric continuals and nominal variables the Spearmans' two-tailed test was used. ROC Curve analysis was used to determine the specificity and sensitivity of sTREM-1 test. For multiple comparison, the Bonferonni correction was used and only a *P* value < 0.005 was considered statistically significant.

## 4. Results

The obtained results can be summarised as follows.

### 4.1. Age of Healthy Subjects and Patients in Different Subgroups of AB

The group of AB patients was divided into the mild intermittent AB, the mild persistent AB, the moderate persistent AB, and the severe persistent AB subgroups. When we analysed age of patients in the particular subgroups, we found asignificant correlation between the age and severity of the disease (Table 1). The results are listed as mean and standard deviation. 

The rest of results are listed as median and interquartile range (IQR).

### 4.2. Comparison of Plasma sTREM-1 between AB Patients and Healthy Controls

**Figure 1:**
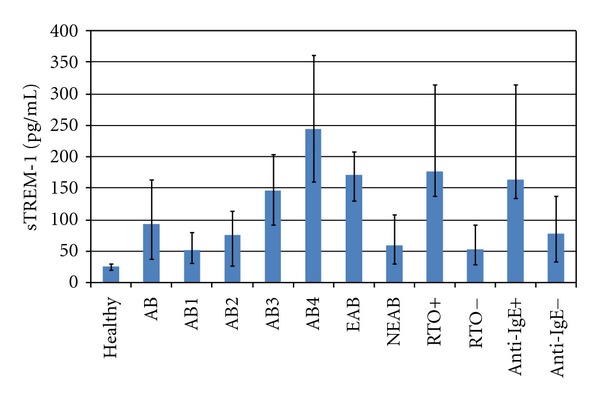
Plasma levels of sTREM-1 in healthy subjects and asthma bronchiale patients AB: asthma bronchiale, AB1: mild intermittent AB, AB2: mild persistent AB, AB3: moderate persistent AB, AB4: severe persistent AB, EAB: exacerbated AB, NEAB: nonexacerbated AB, RTO^+^: asthma with respiratory tract obstruction, RTO^−^: asthma without respiratory tract obstruction, Anti-IgE+: asthma with anti-IgE therapy, Anti-IgE-: asthma without anti-IgE therapy.

 Statistically highly significantly elevated plasma levels of sTREM-1 in AB patients were observed (92.3 pg/mL ± 125.6) compared to those in healthy subjects without AB and any pollen allergy (25.7 pg/mL ± 9.2; *P* = 0.0001, Table 2, Figure 1). Kruskal-Wallis test revealed asignificant association between the plasma levels of sTREM-1 and clinical stages of AB (*P* < 0.005). Plasma levels of sTREM-1 were statistically significantly higher in moderate and severe AB patients comparing to mild intermittent AB patients. 

### 4.3. Comparison of Plasma sTREM-1 between AB Patients with and without Acute Exacerbation

Statistically significantly higher plasma levels of sTREM-1 were found in exacerbating AB patients (EAB) compared to those without AB exacerbation (NEAB): EAB = 170.5 ± 78.2 pg/mL; NEAB = 59.1 pg/mL ± 78.2 pg/mL; *P* = 0.0001, Table 2). The EAB patients experienced also higher numbers of leukocytes (*P* = 0.014) and absolute number of neutrophils (*P* = 0.014, Table 3). However, the relative number of eosinophils were statistically significantly higher in nonexacerbating AB patients (*P* = 0.045). Both, the absolute and relative number of monocytes and lymphocytes were lower in the EAB patients, however, these differences did not reach statistical significance.

### 4.4. Comparison of Plasma sTREM-1 between AB Patients with and without Respiratory Tract Obstruction

Comparing plasma levels of sTREM-1 in the group of AB patients suffering from respiratory tract obstruction (RTO^+^) with those in the RTO negative group (RTO^−^), statistically significant differences were observed (RTO^+^ = 176.4 pg/mL ± 177.8; RTO^−^= 52.0 pg/mL ± 64.0; *P* < 0,0001, Table 2). Patients with respiratory tract obstruction had also higher plasma levels of CRP (*P* = 0.027) and plasma levels of sTREM-1 calculated per one myelocyte (i.e., neutrophil and monocyte, *P* = 0.011) than those without respiratory tract obstruction (Table 4). 

### 4.5. Correlation Test

Spearman's correlation test (Table 5) showed that plasma levels of sTREM-1 significantly correlated with the clinical stage of AB (*P* = 0.0001), the degree of respiratory tract obstruction (*P* = 0.0001), and the presence of anti-IgE omalizumab therapy (*P* = 0.0001). The plasma levels of sTREM-1 in AB patients also significantly correlated with the number of leukocytes (*P* = 0.002) and the absolute number of neutrophils (*P* = 0.001). The correlation of sTREM-1 with increased number of neutrophils belong to rather notable findings. Eventually, the levels of sTREM-1 significantly correlated with those calculated for one myelocyte (*P* = 0.0001).

### 4.6. Sensitivity and Specificity of sTREM-1 Test

**Figure 2:**
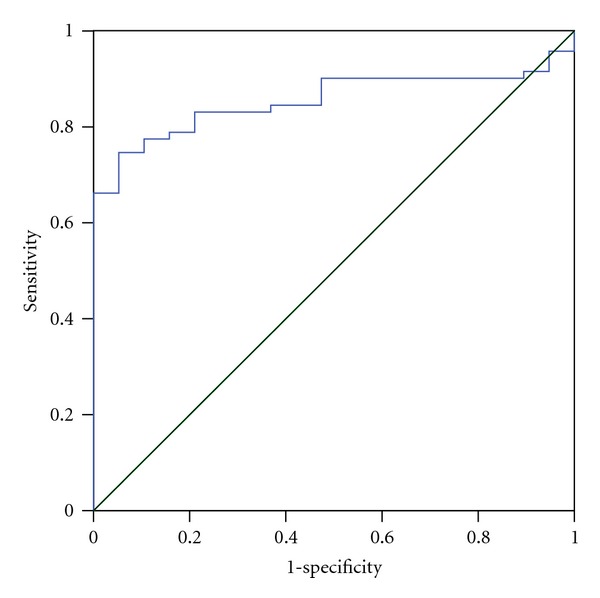
Receiver operating characteristic curve (ROC Curve) of sTREM-1 for asthma bronchiale.

The sensitivity and specificity for sTREM-1 concentrations can be found in Figure 2, and Table 6. The asymptomatic lower bound confidence interval for this test was 0.78 and upper bound confidence interval 0.93. 

## 5. Discussion

Asthma's main features include chronic airways inflammation with a variable degree of air-flow obstruction and bronchial hyperresponsiveness, leading to paroxysmal symptoms of wheeze, cough, shortness of breath, and chest tightness [26]. Antigen-specific Th2 cells and their cytokines IL-4, IL-5, and IL-13 orchestrate the allergic inflammation in asthmatic patients [27–29]. Th17 cells and IL-23 are also involved in antigen-induced airway inflammation, granulopoiesis activation, and neutrophil recruitment mainly in severe asthma, of which neutrophil infiltration is one of the hallmarks [30–33].

In our study, we investigated the plasma levels of a new inflammatory marker sTREM-1 in pollen allergy AB patients. Our results revealed more than 3.5 times higher levels of sTREM-1 in patients with AB compared with healthy subjects without AB and without history of pollen allergy (*P* = 0.0001), indicating for the presence of inflammatory process in asthma patients. The elevated levels of plasma sTREM-1 significantly correlated with clinical stage and severity of AB (Table 2, Figure 1). The highest levels of sTREM-1 were found in patients with moderate and severe persistent AB. The association of the clinical stage of AB patients with their age is documented in Table 1.

We found also significantly higher levels of sTREM-1 in AB patients in exacerbation (EAB) compared with non-exacerbating AB patients (*P* < 0.0001). The group of EAB patients had also significantly higher number of leukocytes (*P* = 0.014) and neutrophils (*P* = 0.014) than NEAB group of patients (Table 3). Respiratory tract obstruction was also followed by elevated levels of sTREM-1 (*P* < 0.0001, Table 2), CRP (*P* = 0.027), leukocytes (*P* = 0.013), and absolute number of neutrophils (*P* = 0.008, Table 4). 

Investigations of plasma levels of sTREM-1 in all subjects of atopic AB patients disclosed the statistically significant correlation of its levels with the clinical stage of AB, respiratory tract obstruction, levels of CRP, the presence of anti-IgE therapy, number of leukocytes, absolute number of neutrophils, and the level of sTREM-1 calculated per one myelocyte, respectively (Table 5).

There are two papers in PubMed concerning the role of membrane TREM-1 in AB [16, 34], however, the levels of sTREM-1 in AB patients have not been studied yet, so we can not compare our results. Elevated levels of sTREM-1 accompanied by increased number of neutrophils in exacerbated asthma bronchiale (Table 3), asthma with respiratory tract obstruction (Table 4), and severe forms of asthma (Table 5) in our group of atopic AB patients support the results and the opinion of the authors that point to an important role of neutrophils in asthma [27, 32, 33]. Although eosinophilic airway inflammation is recognized as an important feature of some patients with chronic stable asthma, neutrophils are the first cells recruited to the site of the allergic reaction and are eliminated by apoptosis during the resolution of the allergic response. Our findings of increased number of eosinophils in AB patients without acute exacerbation supports this fact (Table 3). The presence of neutrophils may influence clinical presentation and has been linked to the development of severe chronic asthma and sudden severe attacks [32]. Our results support this knowledge, increased number of neutrophils that we found in severe forms of asthma underlies this opinion. Moreover, as sTREM-1 is cleaved and released from the surfaces of activated neutrophils and monocytes, elevated plasma levels of sTREM-1 also underline the role of neutrophils in inflammation that accompaines these clinical conditions.

### 5.1. Conclusion

Our results show that the plasma levels of sTREM-1 are highly elevated in severe forms of asthma, reflect the clinical stage of AB, state of exacerbation, respiratory tract obstruction, and correlate with the number of leukocytes, mainly neutrophils. These results point to the role of neutrophils in inflammation that accompanies these clinical conditions. Our results highlight the potential usefulness of the assessment of the soluble form of TREM-1 in plasma of AB patients.

## Figures and Tables

**Table 1:** Characteristics of investigated asthma bronchiale patients.

Patients	Number	(%)	Age (mean ± STD)	*P* (ANOVA)
Healthy subjects	19	100	37.3 ± 13.7	
AB	76	100	37.4 ± 21.4	>0.05
Mild intermittent AB (AB1)	28	36.8	22.0 ± 10.6	
Mild persistent AB (AB2)	18	23.7	29.7 ± 15.7	>0.05^∗^
Moderate persistent AB (AB3)	14	18.4	48.1 ± 26.2	<0.001^∗^
Severe persistent AB (AB4)	16	21.1	55.5 ± 9.7	<0.001^∗^
Exacerbated AB	25	32.9	54.7 ± 19.0	
Nonexacerbated AB	51	67.1	30.0 ± 18.2	<0.0001^∗∗^
Respiratory tract obstruction (RTO^+^)	30	39.5	52.1 ± 19.2	
Without RTO (RTO^−^)	46	60.5	24.7 ± 12.9	<0.001^∗∗^
Anti-IgE therapy	11	14.5	54.1 ± 13.0	
Without anti-IgE therapy	65	85.5	34.6 ± 21.2	<0.001^∗∗^

AB: asthma bronchiale, ^∗^significance between AB2 versus AB1, AB3 versus AB1, AB4 versus AB1, ^∗∗^significance between age of patients in exacerbated versus nonexacerbated asthma, asthma with versus without respiratory tract obstruction, patients with versus without anti-IgE therapy.

**Table 2:** Plasma levels of sTREM-1 in healthy subjects and asthma bronchiale patients.

Patients	Age (years) (mean ± STD)	sTREM-1 (pg/mL) (Median)	sTREM-1 (IQR)	Mann-Whitney (2-tailed) *P*
Healthy subjects	37.3 ± 13.7	25.7	9.2	
AB patients	37.4 ± 21.4	92.3	125.6	<0.0001
Mild intermittent AB (AB1)	22.0 ± 10.6	50.8	49.2	
Mild persistent AB (AB2)	29.7 ± 15.7	75.7	87.7	=0.333^∗^
Moderate persistent AB (AB3)	48.1 ± 26.2	145.6	111.4	=0.001^∗^
Severe persistent AB (AB4)	55.5 ± 9.7	244.0	201.8	<0.005^∗^
Kruskal-Wallis 2-tailed sg.	*P* < 0.005			
EAB	54.7 ± 19.0	170.5	78.2	
NEAB	30.0 ± 18.2	59.1	78.2	<0.0001^∗∗^
Respiratory tract obstruction (RTO^+^)	52.1 ± 19.2	176.4	177.8	
Without RTO (RTO^−^)	24.7 ± 13.0	52.0	64.0	<0.0001^∗∗^
Anti-IgE therapy	54.1 ± 13.0	163.4	180.1	
Without anti-IgE therapy	34.6 ± 21.2	76.9	104.3	<0.0001^∗∗^

AB: asthma bronchiale, STD: standard deviation, IQR: interquartile range, ^∗^significance between AB2 versus AB1, AB3 versus AB1, AB4 versus AB1, EAB: exacerbated asthma bronchiale, NEAB: non-exacerbated asthma bronchiale, RTO^+^: asthma with respiratory tract obstruction, RTO^−^: asthma without respiratory tract obstruction, ^∗∗^significance between exacerbated versus non-exacerbated asthma, asthma with versus without respiratory tract obstruction, patients with versus without anti-IgE therapy.

**Table 3:** Comparison of plasma levels of investigated parameters in asthma bronchiale patients with and without exacerbation.

	sTREM-1 pg/mL	Leu 1·10^6^/mL	Neu (abs) 1·10^6^/mL	Eo (rel) %
EAB	170.5 ± 78.2	10.1 ± 5.9	6.7 ± 4.4	1.3 ± 4.8
NEAB	59.1 ± 78.2	7.4 ± 1.8	4.2 ± 2.4	5.4 ± 6.6
Mann-Whitney 2-tailed sg.	*P* < 0.0001	*P* = 0.014	*P* = 0.014	*P* = 0.045

Leu: leukocytes, Neu: neutrophils, Eo: eosinophils, EAB: exacerbated asthma bronchiale, NEAB: non-exacerbated asthma bronchiale.

**Table 4:** Comparison of plasma levels of investigated parameters in asthma bronchiale patients with and without respiratory tract obstruction.

	sTREM-1 (pg/mL)	CRP mg/mL	Leu 1·10^6^/mL	Neu (abs) 1·10^6^/mL	TREM-1/myelocyte
RTO^+^	176.4 ± 177.8	5.8 ± 7.4	9.0 ± 3.6	5.8 ± 3.6	2.9 ± 2.5
RTO^−^	52.0 ± 64.0	2.5 ± 0.9	6.7 ± 1.4	3.9 ± 1.4	1.8 ± 0.9
Mann-Whitney 2-tailed sg.	*P* < 0.0001	*P* = 0.027	*P* = 0.013	*P* = 0.008	*P* = 0.011

Leu: leukocytes, CRP: C-reactive protein, Neu: neutrophils, Eo: eosinophils, RTO^+^: asthma bronchiale with respiratory tract obstruction, RTO^−^: asthma bronchiale without respiratory tract obstruction.

**Table 5:** Correlations of plasma levels of sTREM-1 with investigated parameters.

	Spearman's correlation test with sTREM-1 (*P*)	Correlation coefficient
Clinical stage of AB	*P* = 0.0001	0.682
Respiratory tract obstruction	*P* = 0.0001	0.653
Anti-IgE therapy	*P* = 0.001	0.421
CRP	*P* = 0.009	0.499
Total level of IgE	*P* = 0.032	0.290
Leukocytes	*P* = 0.002	0.530
Neutrophils (absolute number)	*P* = 0.001	0.579
Neutrophils (relative number)	*P* = 0.044	0.371
Lymphocytes (absolute number)	*P* = 0.013	−0.442
sTREM-1 per one myelocyte	*P* = 0.0001	0.920

AB: asthma bronchiale, CRP: C-reactive protein. Taking into account more correlated parameters, statistically significant are values when *P* < 0.005.

**Table 6:** Sensitivity and specificity of sTREM-1 test. Coordinates of the curve.

sTREM-1 (pg/mL)	Sensitivity	Specificity
2.92	100	0.0
10.4	95.8	0.053
20.6	90.1	15.8
24.9	90.1	47.4
25.8	90.1	52.6
27.9	84.5	63.2
29.6	83.1	73.7
30.3	83.1	78.9
34.6	78.9	84.2
36.3	77.5	84.2
37.5	77.5	94.7
42.8	74.6	94.7
48.4	71.8	94.7
49.8	66.2	100.0
51.0	66.2	100.0
62.7	60.6	100.0
72.1	57.7	100.0
87.5	50.7	100.0
102.9	45.1	100.0
196.6	16.9	100.0
279.5	9.9	100.0
494.0	2.8	100.0
546.1	1.4	100.0
555.4	0.0	100.0

## Abbreviations

AB: Asthma bronchiale
AB1: Mild intermittent asthma
AB2: Mild persistent asthma
AB3: Moderate persistent asthma
AB4: Severe persistent asthma
CRP: C-reactive protein
EAB: Exacerbated asthma bronchiale
IQR: Interquartil range
NEAB: Non-exacerbated asthma bronchiale
RTO^+^: With respiratory tract obstruction
RTO^−^: Without respiratory tract obstruction
STD: Standard deviation
sTREM-1: Soluble Triggering receptor expresssed on myelocytes
TREM-1: Triggering receptor expressed on myelocytes.
